# Massive Carbon Dioxide Embolism During Laparoscopic Liver Resection: A Case Report

**DOI:** 10.7759/cureus.5075

**Published:** 2019-07-03

**Authors:** Alessandro De Cassai, Riccardo Boetto, Giulia Gabellini, Umberto Cillo

**Affiliations:** 1 Anesthesiology, University of Padova, Padova, ITA; 2 Surgical, Oncological and Gastroenterological Sciences, Hepatobiliary Surgery and Liver Transplantation Unit, Padova University Hospital, Padova, ITA; 3 Medicine/ Section of Anesthesiology and Intensive Care, Padova University Hospital, Padova, ITA

**Keywords:** portal vein gas embolism, liver resection, laparoscopy

## Abstract

Carbon dioxide embolism during laparoscopic surgery is a serious and life-threatening complication. The overall incidence of embolism during laparoscopic surgery is low (0.15%). Although the potential fatal consequences of this complication are reported in literature, a well-documented report of the effect of massive CO2 embolism during laparoscopic liver resection on cardiovascular, respiratory and encephalographic parameters does not exist.

The authors describe a well-documented case of massive carbon dioxide embolism during laparoscopic liver resection suspected by both hemodynamic instability and elevation of EtCO2 and confirmed by arterial blood gas. The surgeon's rapid closure of the vascular breach resulted in an overall improvement of the patient’s vital signs without further consequences.

Our case report shows the cardiovascular, respiratory and encephalographic effects of a massive carbon dioxide embolism and highlights the importance of a strict cooperation between the surgeon and the anesthesiologist and the importance for a prompt treatment when massive carbon dioxide embolism occurs.

## Introduction

Laparoscopic liver resection has come to be regarded as a safe surgical procedure for liver disease in selected patients. However, it may be complicated by bleeding and carbon dioxide (CO2) gas embolism (GE), which is less common but potentially life-threatening. CO2 is a gas used to create pneumoperitoneum during video-laparoscopic surgery and, in order to maintain an adequate surgical field, it is continuously insufflated in order to guarantee a constant gas pressure inside peritoneum. GE occurs when gas bubble enters systemic circulation and is a potentially fatal complication of laparoscopic surgery. Fortunately, the overall incidence of GE during laparoscopic surgery is really low (0.15%) but when it develops the incidence of mortality is significant (30%) [1, 2].

We report a case of massive gas embolism during laparoscopic liver resection, promptly resolved without further complications, in order to underline that the most important factor when gas embolism occurs is the time required to repair blood vessel despite the amount of gas embolism occurring. The patient provided written permission for publication of this case report. CARE guidelines were followed while preparing this manuscript [3].

## Case presentation

A 67-year-old woman was admitted to our hospital with a diagnosis of hepatocarcinoma with indication to laparoscopic liver resection of the 8th segment. The patient was an active smoker (one pack/day) with a hepatitis C virus (HCV) infection and had a history of laparotomic cholecystectomy.

Preoperative laboratory findings were normal. On admission at the operatory room, the patient was monitored with (saturimetry, SpO2; heart rate, HR; non-invasive blood pressure; NIBP) and her vitals were stable. An intravenous line (18 gauge) was inserted on her left arm.

After induction of general anesthesia, an endotracheal tube, a central venous catheter (CVC) in the right internal jugular vein and an arterial catheter (20 gauge) in the left radial artery for invasive pressure monitoring were positioned. The patient was then placed in left lateral recumbent position. Controlled mechanical ventilation was initiated using a circle system with carbon dioxide absorber, using a tidal volume of 6 mL/kg and a respiratory rate of 14 breath/min. Usual monitoring was used.

Pneumoperitoneum was induced and maintained at 12 mmHg.

Vital signs kept stable (HR 90 bpm, IBP 100/60, SpO2 98%, etCO2 30 mmHg) with adequate arterial blood gas (pH 7.34, pO2 144.9 mmHg, p/F, SatO2 98.5%, pCO2 27.8 mmHg, PaCO2-etCO2 2.2 mmHg) until deeper plane of parenchymal transection. At this point, etCO2 suddenly increased from 30 to 50 associated with a decrease in SpO2 (85%) and in blood pressure (from 100/60 mmHg to 70/40 mmHg) that occurred rapidly; FiO2 was immediately raised to 100%. A CO2 embolism was suspected and it was confirmed by an arterial blood gas test (pH 7.13, pO2 113.2 mmHg, p/F, SatO2 96%, pCO2 107.5 mmHg, HCO3 37.7 mmol/L). Pneumoperitoneum was lowered to 9 mmHg and PEEP was raised to 9 cm H2O to equalize intra-abdominal pressure and to avoid further embolism. However, embolism continued for few seconds (etCO2 60 mmHg, PaCo2-etCO2 50 mmHg) until a lesion of right hepatic vein collateral was identified, clamped and sutured with vascular clips avoiding further embolism. Laparotomic conversion was not needed. During embolism time, surgeons were able to aspirate only minimal air bubbles from distal CVC line.

Repeated arterial blood analysis was executed showing a rapidly decrease in both PaCO2 and PaCO2-etCO2 with complete normalization in less than an hour.

**Figure 1:**
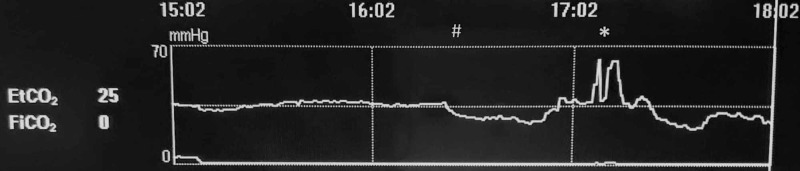
EtCO2 trend # Surgery started * Gas embolism

We are reporting the trend of EtCO2 (Figure 1).

Soon after the operation, the patient was admitted in the intensive care unit and extubated few hours later. Postoperative course underwent uneventful and the patient was discharged from hospital on postoperative day three, without further complications.

## Discussion

Gas embolism during laparoscopic surgery of the liver is a serious and not so uncommon complication, but usually gas emboli are minimal and the only clinical sign detected could be bubbles at echocardiography [4,5].

A literature review by Otsuka et al. on 1262 patients undergoing laparoscopic liver resection showed that only three (0.2%) of them developed gas embolism, two of them developed only a transient lowering in EtCO2 while the third one developed only a transient lowering in blood pressure [4, 6, 7]. Other than that, gas embolism has been only anecdotally reported and diagnosed by paradoxical cerebral embolism [8,9].

When CO2 bubbles enter into pulmonary circulation, their elimination depends on reabsorption in the adjacent structures: primarily the alveoli and, in a lesser degree, blood and adjacent tissues. Until the end of this process, air trapping in pulmonary circulation causes a stop in the pulmonary blood flow resulting in a) rise of the ventilatory dead space that leads to acute hypoxemia and hypercapnia, b) acute increase in pulmonary vascular resistance with acute right ventricular strain, leading to right heart failure and possible circulatory collapse. Moreover, air accumulation in the ventricle does not permit an adequate diastolic filling and there is the possibility that, during systole, air is pumped into the coronary arteries [10].

CO2 has three major ways to be eliminated: a) diffusion, b) transformation into carbonic acid and c) binding to proteins. In particular, a) CO2 is a highly diffusible gas being 20 times more soluble than oxygen (0.0308 mmol/litre*mmHg-1), b) carbon dioxide forms carbonic acid when combined with water, this is a relatively slow reaction per se, however, carbon dioxide diffuses freely in the blood cell combining rapidly with water to form carbonic acid, a reaction that is accelerated by carbonic anhydrase becoming one of the fastest reactions known in the human body, c) carbon dioxide binds to proteins combining to the terminal uncharged amino groups (especially reduced hemoglobin).

Our case depicts a massive gas embolism (PaCO2 raised at 60 mmHg, PaCO2-etCO2 raised from three to 50, hemodynamic instability). The predisposing factors were positive intraperitoneal pressure, low central venous pressure (CVP) and left recumbent position with hepatic veins higher than heart. However, despite increment of PEEP levels, gas embolism stopped only at surgical repair of the vein. This shows, as suggested by Fors et al., that there has to be a mechanism irrespective of difference in IAP-CVP pressure, in which the rhythmic compression and decompression of the ventilation could play a role as a Venturi-like effect [11].

Another interesting effect is the quick decrease in BIS level during gas embolism from 40 to 10. We believe that sudden increase of CO2 to 110 mmHg could have led to an important cerebral vasodilatation with a consequent temporary increase in intracerebral pressure.

However, interestingly immediately after surgical repair of the breach BIS returned to previous numeric values.

## Conclusions

Our case brings with it an important learning point: prognosis in a massive CO2 embolism is probably primarily depending on the timing until surgical closure of the lesion, despite huge amount of CO2 entering in the vascular system. As witnessed by arterial blood gases, hemodynamic and ventilator parameters, CO2 was then promptly reabsorbed with no need of circulatory or ventilatory support in the postoperative period.

We want to stress the importance of both awareness regarding CO2 embolism during laparoscopic hepatic surgery and communication inside the operating room between surgeon and anesthesiologist in order to identify the pivotal moments of vessel exposition and quickly recognize such a potentially fatal complication.
